# Protective effects of docosahexaenoic acid combined with bilberry extract on myopic Guinea pigs

**DOI:** 10.3389/fmed.2024.1502612

**Published:** 2024-12-17

**Authors:** Tainan Lin, Jianzhang Hu, Qian Wen, Xiaoting Liu, Jinghua Lin, Qiaomei Shi, Miao Lin, Weifu Huang

**Affiliations:** ^1^Department of Ophthalmology, Fujian Medical University Union Hospital, Fuzhou, China; ^2^Department of Ophthalmology, Fujian Provincial Governmental Hospital, Fuzhou, China; ^3^TowardPi Medical Technology Beijing, Beijing, China

**Keywords:** docosahexaenoic acid, bilberry extract, lens-induced myopia, choroidal thickness, choroidal vascularity index, Guinea pigs

## Abstract

This study aims to investigate the protective effects of docosahexaenoic acid (DHA) combined with bilberry extract (BE) on myopic guinea pigs. In total, 105 healthy pigmented guinea pigs aged 2 weeks were selected and randomly divided into five groups. The normal control (NC) group received no treatment, while the experimental groups wore −6.0D lenses on the right eye to establish an animal model of lens-induced myopia (LIM). These groups were further divided based on different treatments: normal feeding, DHA treatment, BE treatment, and combined DHA + BE treatment. Refractive error and axial length for both eyes were measured before modeling, after 4 weeks of modeling, and after 8 weeks of treatment. Fundus examination was performed, and choroidal thickness, choroidal vascularity index (CVI), maximal mixed response in dark adaptation (Max-ERG), and cone cell response in light adaptation (Cone-ERG) were measured. After 8 weeks of treatment, we observed a significant reduction in refractive error and shortening of axial length, improvement in fundus condition, and increased choroidal thickness and CVI in the LIM + DHA + BE group. Electroretinogram (ERG) showed that the amplitudes of a-wave and b-wave were enhanced in both Max-ERG and Cone-ERG tests. The LIM + DHA + BE group exhibited superior effects compared to the LIM + DHA group and the LIM + BE group. The combination of DHA and BE delayed the progression of LIM in guinea pigs and was more effective than DHA or BE alone. The synergistic effect of DHA and BE offers a new approach to the prevention and treatment of myopia.

## 1 Introduction

In recent years, the incidence of myopia has progressively increased among children in China, with early-onset myopia being particularly prominent (1, 2). Consequently, the prevention and treatment of myopia have become top priorities. Currently, myopia is primarily managed through behavioral, pharmaceutical, and optical interventions, each with its limitations. Behavioral interventions, such as increased outdoor activity time, can effectively prevent myopia, but it is challenging to ensure and maintain consistent outdoor activity (3, 4). Pharmaceutical interventions, such as the use of low-concentration atropine drops, have been effective in slowing the progression of myopia in some children; however, side effects like photophobia and increased intraocular pressure have limited their use (5, 6). Additionally, orthokeratology lenses pose an increased risk of infectious keratitis and necessitate professional support (7, 8). Our previous study also demonstrated a close relationship between choroidal thickness (ChT) and both blood flow and myopia (9). Studies have indicated that reduced choroidal blood perfusion (ChBp) is a crucial factor involved in scleral hypoxia and the development and progression of myopia (10, 11). Therefore, improving scleral oxygenation may be an effective treatment strategy for controlling the progression of myopia (12). In the absence of effective treatment methods, dietary supplements have received increasing attention as safe and feasible means of controlling and preventing myopia (13–17).

Docosahexaenoic acid (DHA) is an essential dietary fatty acid for the human body (18, 19). Studies have shown that dietary DHA protects against myopia (20, 21), and lipidomic analysis has demonstrated that DHA inhibits choroidal thinning associated with myopia (22). These animal and human studies suggest that DHA is an accessible candidate for controlling myopia. However, children often do not consume sufficient dietary DHA, necessitating dietary supplementation (11, 23).

Previous studies have found that bilberry extract (BE) promotes the regeneration of rhodopsin, improves retinal microcirculation, and enhances night vision (24–26). However, whether BE can delay the progression of myopia by acting on choroidal blood vessels remains unclear. Recent studies have combined BE with DHA to form a mixture, finding that their synergistic effects enhance their therapeutic benefits, more effectively alleviating eye fatigue and improving fundus blood flow (27, 28). Therefore, this study aimed to explore the combined effects of DHA and BE in controlling myopia, offering new ideas and methods for myopia prevention and control.

## 2 Materials and methods

### 2.1 Animal husbandry and grouping

In total, 105 two-week-old male British tricolor shorthair guinea pigs (110 ± 10 g) were purchased from Jiangxi Zhonghong Boyuan Biotechnology Co., Ltd. (License No. SYXK-Gan2020-0001). The guinea pigs were housed at the Animal Experiment Center of the Fujian Provincial Center for Disease Control and Prevention. The guinea pigs were kept in transparent clean plastic cages, with 4–5 guinea pigs per cage. The lighting intensity was approximately 300 lux, with an ambient temperature of 22°C. The guinea pigs had free access to water and food under natural light, strictly adhering to a 12 h (h) /12 h light–dark cycle. All guinea pigs underwent an ocular examination to exclude conditions such as congenital myopia, cataracts, and corneal diseases. After 1 week of acclimatization, the guinea pigs were randomly divided into five groups: normal control (NC), lens-induced myopia (LIM), DHA-treated LIM (LIM + DHA), BE-treated LIM (LIM + BE), and DHA + BE-treated LIM (LIM + DHA + BE), with 21 guinea pigs in each group. We take the method of random number grouping for 105 guinea pigs. Firstly, we prepared paper strips, wrote the numbers from 1 to 105 on 105 paper strips, and marked the guinea pigs’ ear tags with the numbers from 1 to 105. Then lots were drawn and the slips of paper were placed into a closed container and 21 slips of paper were randomly selected for the first group, followed by 21 slips of paper for the next group, and so on until all groups were complete. Finally, confirm the grouping, ensuring that each group has the same number of samples, and record the guinea pig number of each group for subsequent experiments. After completing the grouping, the guinea pigs in each group can be experimented on according to the experimental design, keeping the other variables consistent to ensure the reliability of the results. The feeding temperature was 20–26°C, humidity was 40–70%, light level was 200–400 Lux, light/dark time ratio was 12/12 h per day, and the guinea pigs were fed with specialized guinea pig feeds with sufficient water and feed, and the guinea pigs were supplemented with appropriate amount of vitamin C in the water. All animal experimental procedures followed the norms for the use of animals in ophthalmic research as set out in the American Association for Research in Vision and Ophthalmology (ARVO) declaration. The management of the experimental animals complied with the “Regulation on the Administration of Laboratory Animals of the Ministry of Health of the People’s Republic of China” and was approved by the ethics committee of Fujian Provincial Governmental Hospital, (Ethical Approval Number: RL2024-04).

### 2.2 Establishment of the LIM model

**Figure 1:**
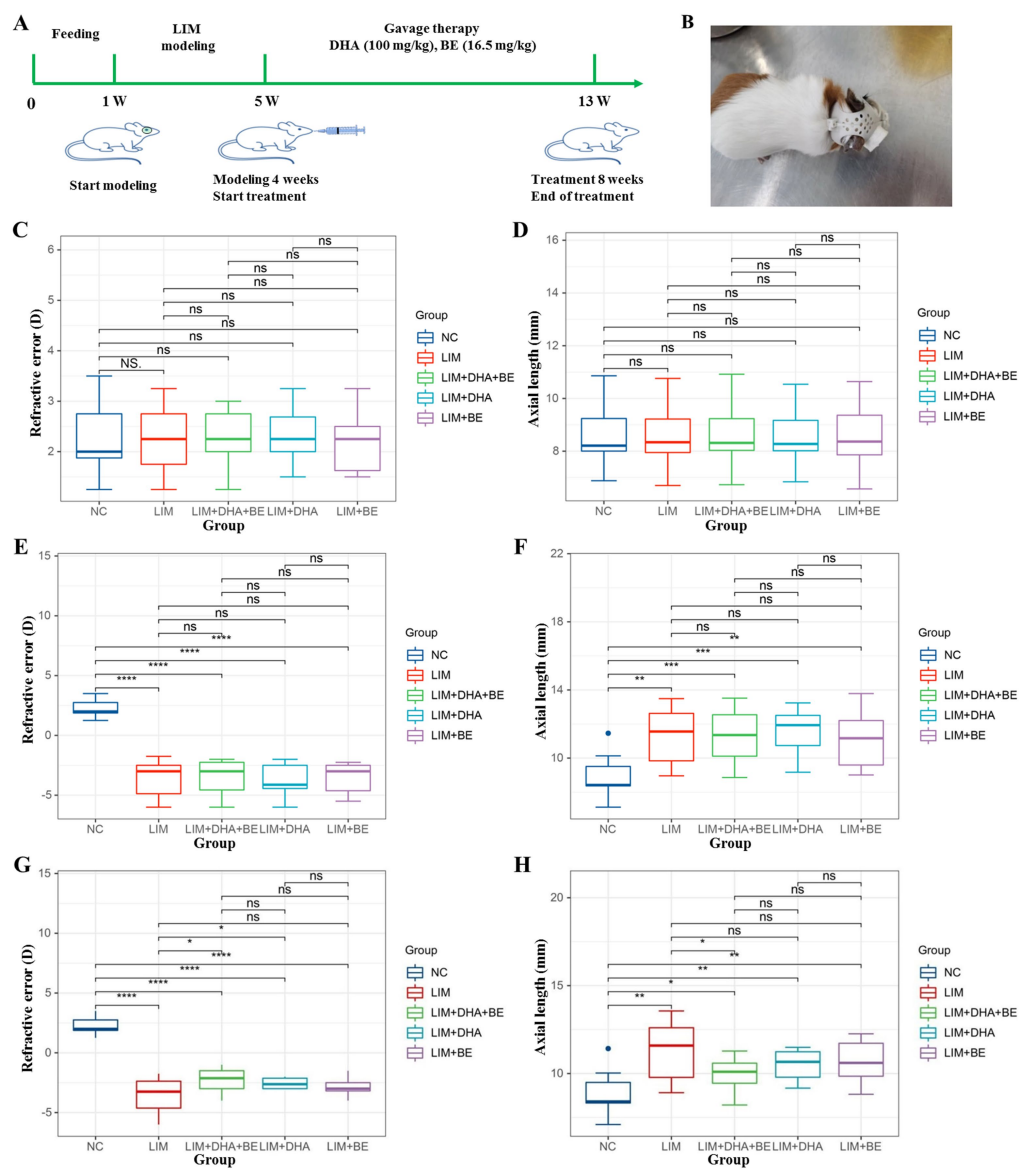
Changes in refractive error and axial length in guinea pigs before and after the construction and treatment of the LIM model. **(A)** Schematic of the LIM model construction and treatment process; **(B)** Guinea pig with PMMA lens on the right eye in the LIM model; **(C)** Comparison of refractive error results among groups before modeling; **(D)** Comparison of axial length results among groups before modeling; **(E)** Comparison of refractive error results among groups 4 weeks after modeling; **(F)** Comparison of axial length results among groups 4 weeks after modeling; **(G)** Comparison of refractive error results among groups 8 weeks after treatment; **(H)** Comparison of axial length results among groups 8 weeks after treatment. LIM: Lens-induced myopia; PMMA: Polymethyl methacrylate; NC: Normal control; LIM: Lens-induced myopia; DHA: Docosahexaenoic acid; BE: Bilberry extract. ns *p* > 0.05, *p < 0.05, **p < 0.01, ***p < 0.001, ****p < 0.0001.

The animal model of LIM was prepared using a defocus-induced myopia modeling method. Custom −6.0D polymethyl methacrylate (PMMA) lenses were used, with the right eye fitted with a −6.0D lens and the left eye uncovered as the internal control. During the study, the eyes of guinea pigs were examined daily. Apart from the NC group, guinea pigs in the negative control and pharmaceutical intervention groups wore a 3D printed headgear fitted with PMMA lenses on the right eye for 4 weeks, inducing defocus-type myopia in the right eye. The LIM model was evaluated based on changes in refractive error and axial length. Increased myopic refractive error and elongation of the axial length after modeling indicated the successful establishment of the model (Figures 1A,B).

### 2.3 Treatment methods

After the successful establishment of the model, the groups underwent gastric lavage treatment with medications for 8 weeks. The NC group and the LIM group received normal food according to the results of the equivalent dose conversion and pre-test. The LIM + DHA group received gastric lavage with DHA algal oil (containing 35% DHA) at a dose of 100 mg/kg, which was five times the recommended human dose of 20 mg/kg (21). The LIM + BE group received gastric lavage with BE at a dose of 16.5 mg/kg, which was five times the recommended human dose of 3.3 mg/kg. BE solid powder (extracted with 70% ethanol) was dissolved in edible vegetable oil (29). The LIM + DHA + BE group received gastric lavage with DHA algal oil (containing 35% DHA) at a dose of 100 mg/kg combined with BE solid powder (extracted with 70% ethanol) at a dose of 16.5 mg/kg. Given the viscous, oily nature of the mixture, it was diluted with edible oil before administration (Figure 1A). The process of preparation of DHA and BE extracts is described in Supplementary materials.

### 2.4 Measurement of refractive error and axial length

Before modeling, 4 weeks after modeling, and 8 weeks after treatment, the refractive error of each group of guinea pigs was measured using streak retinoscopy. Before the examination, pupils were dilated with 1% cyclopentolate hydrochloride eye drops. Two drops were administered every 10 min. Refractive measurements were performed 30 min later, with a working distance of 50 cm. The entire process was conducted by the same skilled optometrist in a dark room, averaging the refractive values from the vertical and horizontal principal meridians. Axial length measurements were conducted using an ophthalmic A-scan ultrasonography device (Tianjin Suowei Electronic Technology Co., Ltd.). Before measurement, the surface of the eye was anesthetized with 0.5% proparacaine. During the measurement, the probe was lightly touched to the corneal surface and positioned perpendicular to the corneal apex. Manual continuous measurements were taken 10 times. The outliers were removed, and the average value was calculated. The entire procedure was completed by the same skilled technician.

### 2.5 Measurement of ChT and CVI in guinea pigs

BMizar 400KHz Full-Range SS-OCT (TowardPi Medical Technology Co., Ltd., Beijing, China) was used to measure the guinea pigs’ ChBP. The pupils of each guinea pig were dilated using 1% cyclopentolate hydrochloride eye drops. Under dim red light, the guinea pigs were anesthetized intraperitoneally with 3% pentobarbital sodium (50 mg/kg). Blood flow was captured using the optical coherence tomography angiography (OCTA) mode, with scans centered on the optic disk. The scans were conducted horizontally and vertically, covering an area of 12 × 12 mm. Each position was scanned twice to obtain optimal images. The optical media of guinea pig eyes were clear, and the quality of the OCTA images reached a score of 10. Images with a quality score of 6 or higher were considered acceptable. After completing these measurements, ChT was analyzed by the software to detect the CVI of the guinea pigs.

### 2.6 Electroretinogram measurement

We utilized the Celeris system (Diagnosys LLC) electrophysiological operating system to assess changes in guinea pig visual function. ERG measurements primarily recorded three types of electrophysiological responses on the retina: maximal mixed response in dark adaptation (Max-ERG), cone cell response in light adaptation (Cone-ERG), and oscillatory potentials in both dark and light adaptation (OPS). ERG analysis was used to determine the latency and amplitude of each waveform. The guinea pigs were dark-adapted for 12 h beforehand, and their pupils were dilated with 1% cyclopentolate hydrochloride eye drops 30 min before recording. Under dim red light, the guinea pigs were anesthetized with an intraperitoneal injection of 3% pentobarbital sodium (50 mg/kg) and laid flat on a platform with fixed support. A heating pad was used to maintain the body temperature of guinea pigs. Their eyes were anesthetized with 0.5% proparacaine and protected with levofloxacin. Ag/AgCl corneal electrodes were placed on both eyes, and the guinea pigs received rapid stimuli through the corneal stimulator. The corneal stimulator was carefully aligned with the corneal surface to minimize contact errors during measurements.

### 2.7 Statistical analysis methods

All data were analyzed using SPSS version 22.0 (IBM, Armonk). The Shapiro–Wilk normality test was used to assess the distribution of data. Independent data were compared using unpaired two-tailed student’s t-tests or nonparametric Mann–Whitney U tests. Optometry was included. For multiple comparisons, one-way analysis of variance (ANOVA) or two-way ANOVA with Bonferroni’s *post hoc* tests were utilized. Several types of data were included: axial measurements, ChT and CVI Max-ERG, OPS, and Cone-ERG. *p* < 0.05 indica una differenza statisticamente significativa, ns *p* > 0.05, *p < 0.05, **p < 0.01, ***p < 0.001, ****p < 0.0001.

## 3 Results

### 3.1 Changes in refractive error and axial length

Before modeling, there were no significant differences in refractive error and axial length between the groups (Figures 1C,D). Four weeks after modeling, the myopic refractive error of the right eye significantly increased in the LIM (−3.65 ± 1.52 D), LIM + DHA + BE (−3.50 ± 1.43 D), LIM + DHA (−3.70 ± 1.27 D), and LIM + BE (−3.55 ± 1.27 D) groups compared to the NC group (2.27 ± 0.68 D) (Figure 1E; *p* < 0.001). The axial length of the right eye also significantly increased in the LIM (11.33 ± 1.68 mm), LIM + DHA + BE (11.27 ± 1.59 mm), LIM + DHA (11.53 ± 1.40 mm), and LIM + BE (11.53 ± 1.40 mm) groups compared to the NC group (8.61 ± 1.05 mm) (Figure 1F; *p* < 0.01). After 8 weeks of treatment, the myopic refractive error of the right eye decreased in the LIM + DHA + BE (−2.33 ± 0.99 D) and LIM + DHA (−2.58 ± 0.44 D) groups compared to the LIM group (−3.64 ± 1.48 D) (Figure 1G; *p* < 0.05). The axial length of the right eye in the LIM + DHA + BE group (9.94 ± 1.09 mm) showed a significant reduction compared to the LIM group (11.31 ± 1.71 mm) (*p* < 0.05), while there were no significant differences between the LIM + DHA group (10.42 ± 1.06 mm) and the LIM + BE group (10.59 ± 1.57 mm; (Figure 1H). There were no significant differences in the refractive error and axial test results of the left eyes of guinea pigs in all groups at 4 weeks after modeling and 8 weeks after treatment (Supplementary Figures S1A–D).

### 3.2 Changes in ChT and CVI of guinea pigs

**Figure 2:**
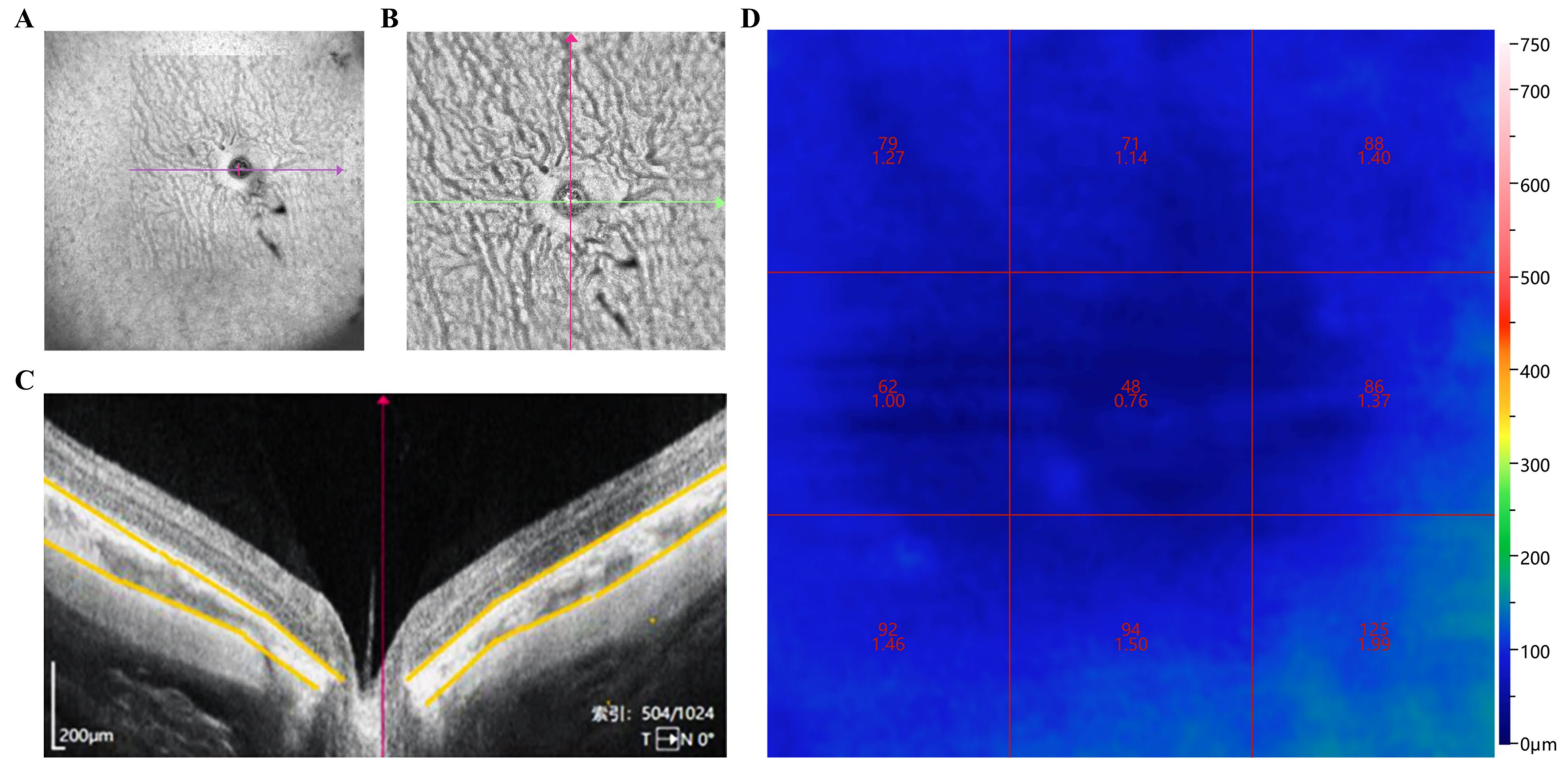
Assessment of ChT and CVI changes in guinea pigs. **(A)** Wide-angle view of the retinal blood flow in guinea pig eyes; **(B)** Blood flow around the optic nerve and peripheral retina; **(C)** ChT centered on the optic disk (area marked by yellow line); **(D)** CVI in nine regions centered on the optic disk. ChT: Choroidal thickness; CVI: Choroidal vascularity index.

**Figure 3:**
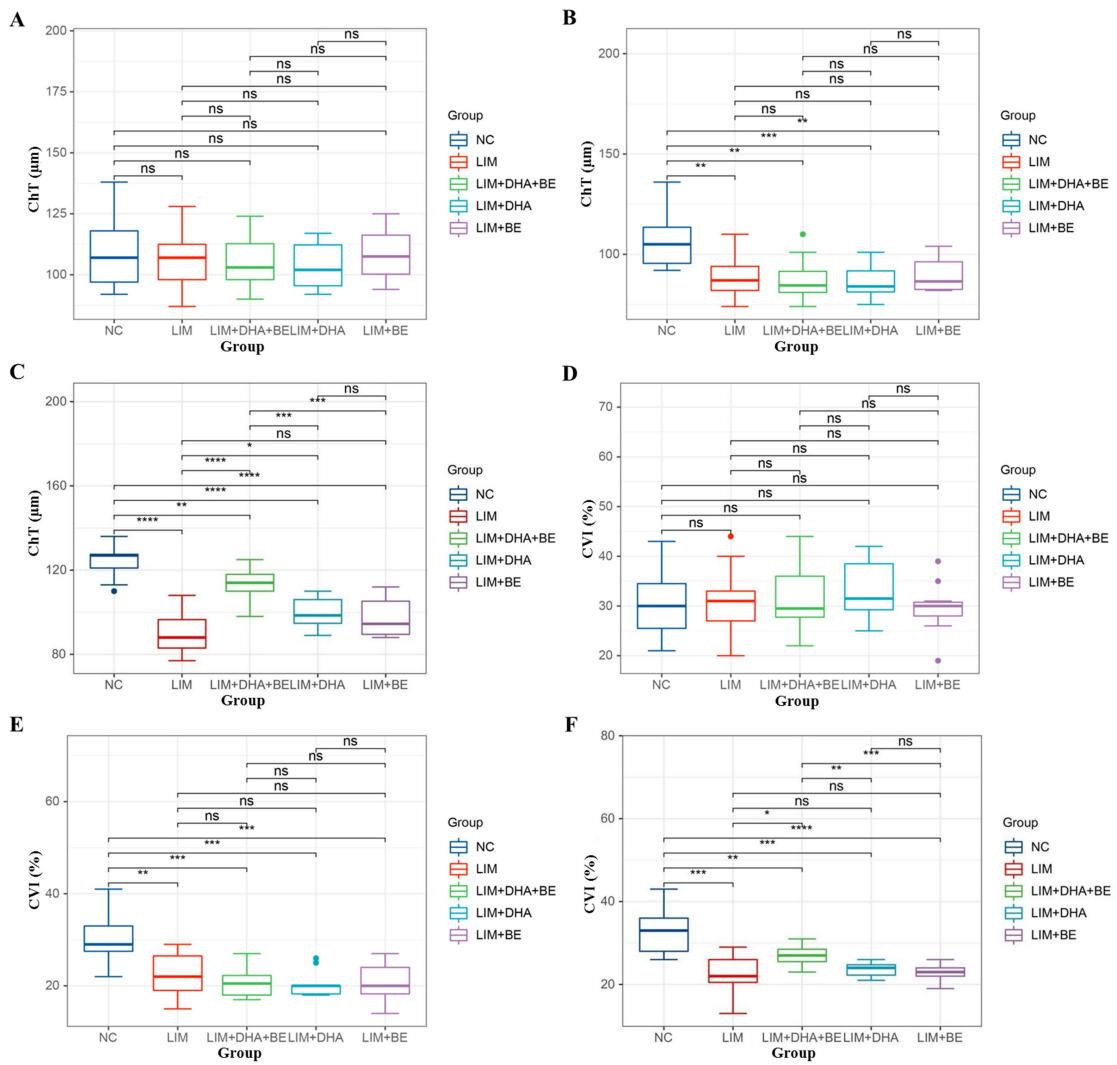
Comparison of ChT and CVI results in guinea pigs. **(A)** Comparison of ChT across groups before modeling; **(B)** Comparison of ChT across groups 4 weeks after modeling; **(C)** Comparison of ChT across groups 8 weeks after treatment; **(D)** Comparison of CVI across groups before modeling; **(E)** Comparison of CVI across groups 4 weeks after modeling; **(F)** Comparison of CVI across groups 8 weeks after treatment. ChT: Choroidal thickness; CVI: Choroidal vascularity index. ns *p* > 0.05, *p < 0.05, **p < 0.01, ***p < 0.001, ****p < 0.0001.

We assessed the ChT and right eye CVI of guinea pigs in all groups (Figures 2A–D). Before modeling, there were no significant differences in ChT among the groups (Figure 3A). Four weeks after modeling, the right eye ChT significantly decreased in the LIM (89.00 ± 10.37 μm), LIM + DHA + BE (87.5 ± 9.97 μm), LIM + DHA (86.80 ± 8.38 μm), and LIM + BE (89.8 ± 8.57 μm) groups compared to the NC group (107.20 ± 12.48 μm; Figure 3B; *p* < 0.001). After 8 weeks of treatment, compared to the LIM group (89.00 ± 10.37 μm), the right eye ChT showed no significant change in the LIM + BE group (97.30 ± 9.52 μm), increased in the LIM + DHA group (94.07 ± 110.25 μm), and significantly increased in the LIM + DHA + BE group (98.08 ± 9.62 μm; Figure 3C; Supplementary Table S1, *p* < 0.001). Before modeling, there were no significant differences in the right eye CVI among the groups (Figure 3D). Four weeks after modeling, the right eye CVI significantly decreased in the LIM (22.55 ± 4.59%), LIM + DHA + BE (20.75 ± 3.31%), LIM + DHA (20.40 ± 2.84%), and LIM + BE (20.80 ± 4.08%) groups compared to the NC group (30.64 ± 5.97%) (Figure 3E; *p* < 0.001). After 8 weeks of treatment, compared to the LIM group (22.73 ± 4.43%), there were no significant changes in the right eye CVI in the LIM + DHA (24.07 ± 3.62%) and LIM + BE (23.70 ± 3.89%) groups, but an increase was observed in the LIM + DHA + BE group (27.17 ± 5.46%) (Figure 3F; Supplementary Table S1; *p* < 0.05). In the right eye of the NC group, the central area of the fundus was seen to be covered by the intact choroid, and the larger blood vessels below were completely covered by capillaries and small vessels, which could not be clearly identified in the color fundus images even under the strongest light intensity. Compared with the NC group, the fundus of the right eye in the LIM group, LIM + DHA group, LIM + BE group, LIM + DHA + BE group at 4 weeks after modeling showed larger blood vessels around the optic disk and thin sheets of capillaries, small blood vessels, and small areas without blood vessels were seen in the larger blood vessels, and choroidal atrophy, and in severe cases, the choroidal and small vessel layers were seen to be completely lost, leaving behind only twisted and enlarged blood vessels. Compared with 4 weeks after modeling, choroidal atrophy was seen to be reduced in the fundus of the LIM + DHA group at 12 weeks after treatment, and some of the gross choroidal and small vessel layers were restored (Figure 3). The ChT and CVI test results in the left eyes of guinea pigs in all groups were not significantly different at 4 weeks after modeling and 8 weeks after treatment (Supplementary Figures S2A–D).

### 3.3 Results of max-ERG, OPS, and cone-ERG measurements

**Figure 4:**
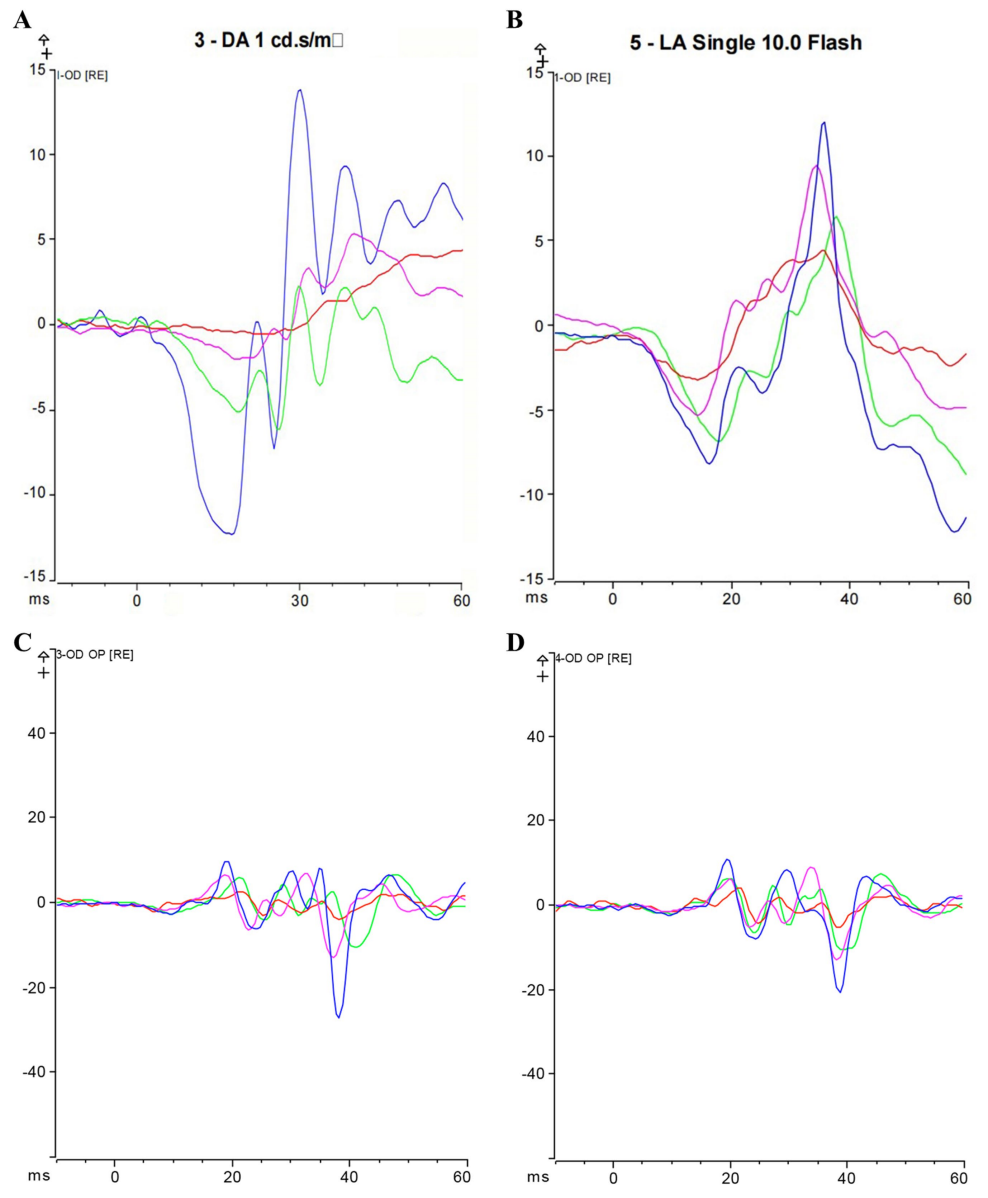
Max-ERG and Cone-ERG results after 8 weeks of treatment; **(A)** Overlaid a-wave and b-wave forms of Max-ERG across groups; **(B)** Overlaid a-wave and b-wave forms of Cone-ERG across groups; **(C)** Guinea pig dark-adapted OPS recording; **(D)** Guinea pig light-adapted OPS recording. Blue represents the LIM + DHA + BE group, purple represents the LIM + DHA group, green represents the LIM + BE group, red represents the LIM group. Max-ERG: Maximal mixed response in dark adaptation; Cone-ERG: Cone cell response in light adaptation; OPS: oscillatory potentials in both dark and light adaptation.

**Figure 5:**
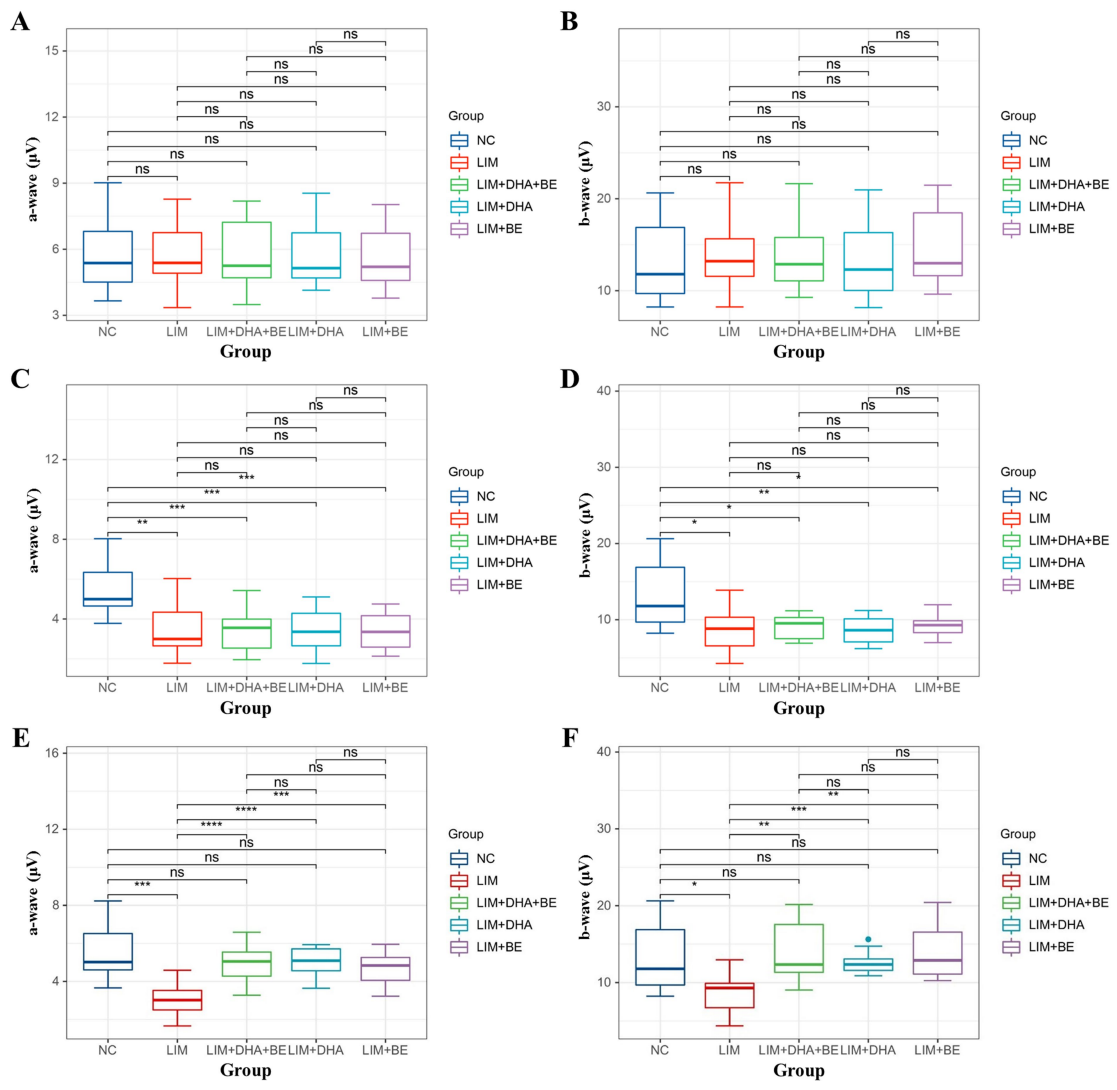
Comparison of a-wave and b-wave results in Max-ERG; **(A)** Comparison of a-wave results in Max-ERG among groups before modeling; **(B)** Comparison of b-wave results in Max-ERG among groups before modeling; **(C)** Comparison of a-wave results in Max-ERG among groups 4 weeks after modeling; **(D)** Comparison of b-wave results in Max-ERG among groups 4 weeks after modeling; **(E)** Comparison of a-wave results in Max-ERG among groups after 8 weeks of treatment; **(F)** Comparison of b-wave results in Max-ERG among groups after 8 weeks of treatment. Max-ERG: Maximal mixed response in dark adaptation; NC: Normal control; LIM: Lens-induced myopia; DHA: Docosahexaenoic acid; BE: Bilberry extract. ns *p* > 0.05, *p < 0.05, **p < 0.01, ***p < 0.001, ****p < 0.0001.

**Figure 6:**
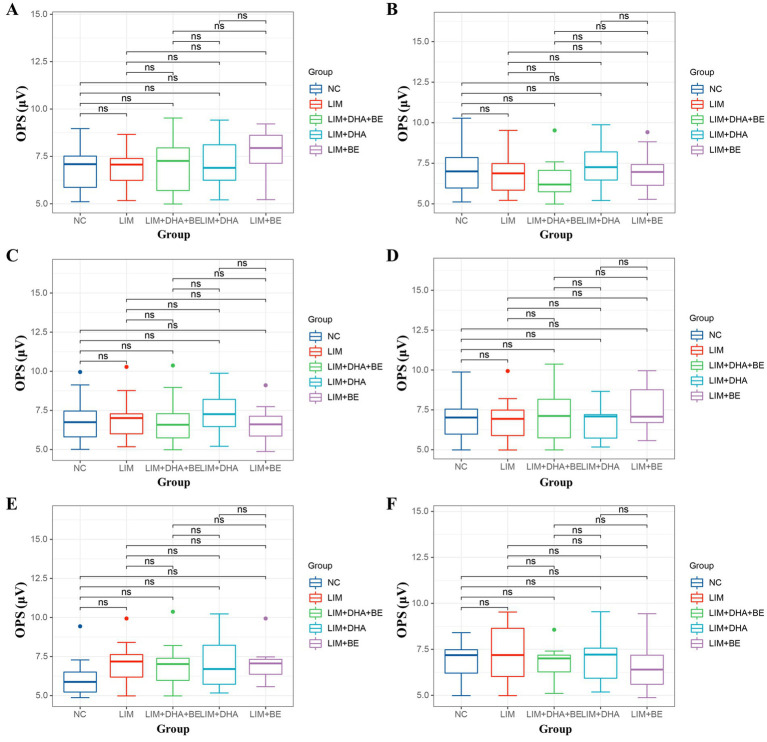
Guinea pig dark-adapted and light-adapted OPS measurement results. **(A)** Dark-adapted OPS measurement results among groups before modeling; **(B)** Dark-adapted OPS measurement results among groups 4 weeks after modeling; **(C)** Dark-adapted OPS measurement results among groups after 8 weeks of treatment; **(D)** Light-adapted OPS measurement results among groups before modeling; **(E)** Light-adapted OPS measurement results among groups 4 weeks after modeling; **(F)** Light-adapted OPS measurement results among groups after 8 weeks of treatment. OPS: oscillatory potentials in both dark and light adaptation; NC: Normal control; LIM: Lens-induced myopia; DHA: Docosahexaenoic acid; BE: Bilberry extract. ns *p* > 0.05.

We assessed Max-ERG and Cone-ERG in all groups of guinea pigs (Figures 4A–D). The results of Max-ERG indicated that before modeling, under dark-adapted conditions with LA 1 cd*s/m2 stimulation, there were no significant differences in the a-waves and b-waves of Max-ERG among the groups (Figures 5A,B). Four weeks after modeling, compared to the NC group (a-wave 5.37 ± 2.05 μV; b-wave 11.34 ± 3.78 μV), the right eye Max-ERG a-waves and b-waves were reduced in the LIM (a-wave 3.26 ± 1.87 μV; b-wave 8.76 ± 2.87 μV), LIM + DHA + BE (a-wave 3.42 ± 1.07 μV; b-wave 8.52 ± 1.70 μV), LIM + DHA (a-wave 3.38 ± 1.14 μV; b-wave 8.61 ± 1.76 μV), and LIM + BE (a-wave 3.38 ± 0.91 μV; b-wave 8.81 ± 1.50 μV) groups (Figures 5C,D, a-wave, *p* < 0.01; b-wave, *p* < 0.05). After 8 weeks of treatment, compared to the LIM group (a-wave 3.36 ± 1.41 μV; b-wave 8.79 ± 2.73 μV), the Max-ERG a-waves and b-waves of the right eye increased in the LIM + DHA + BE (a-wave 5.11 ± 0.88 μV; b-wave 14.09 ± 3.99 μV), LIM + DHA (a-wave 5.05 ± 0.75 μV; b-wave 12.63 ± 1.63 μV), and LIM + BE (a-wave 4.71 ± 0.87 μV; b-wave 13.96 ± 3.42 μV) groups (Figures 5E,F, a-wave, *p* < 0.001; b-wave, *p* < 0.01). Additionally, there were no significant differences in dark-adapted OPS waves before modeling, 4 weeks after modeling, and 8 weeks after treatment (Figures 6A–C).

**Figure 7:**
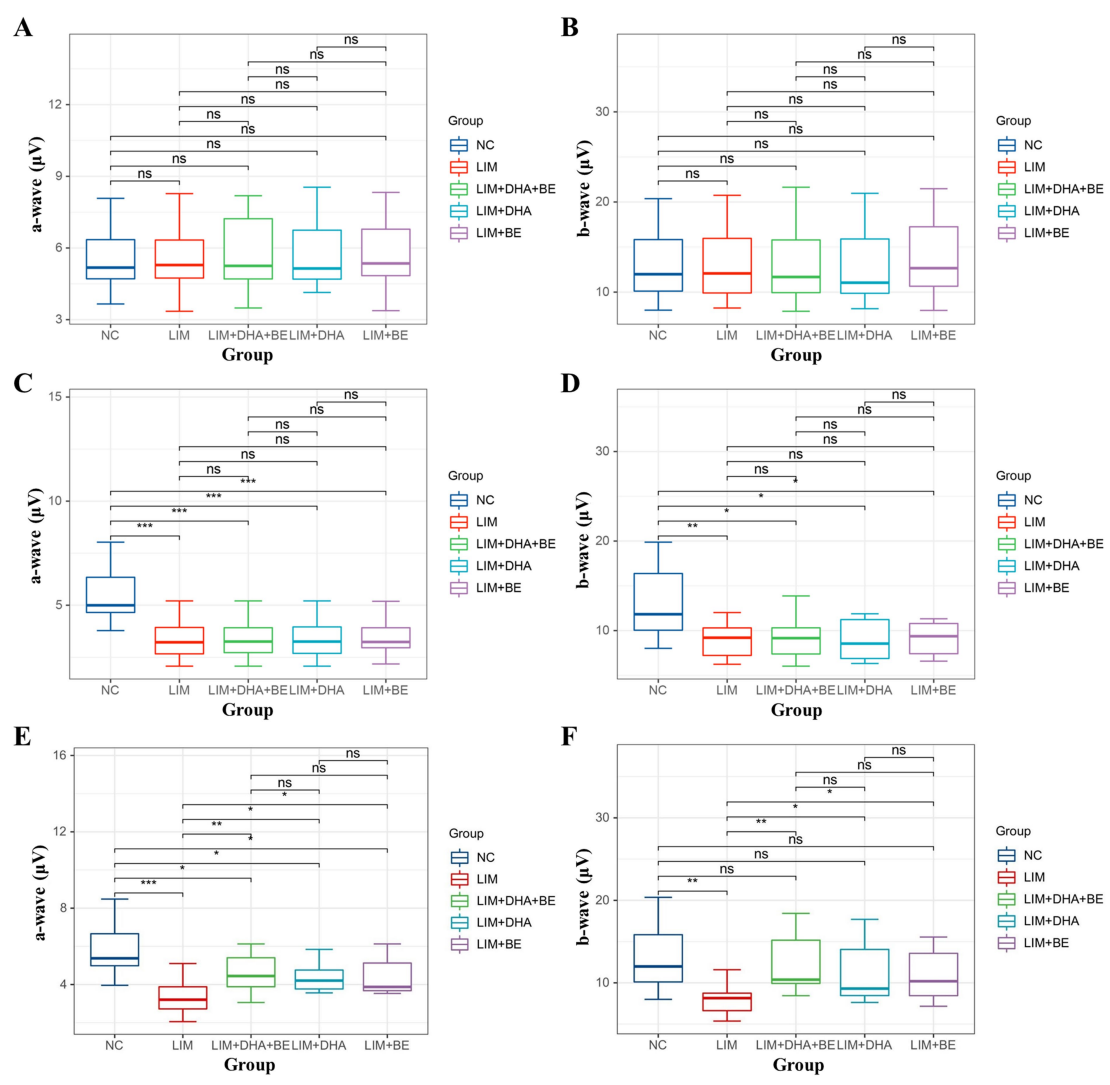
Comparison of a-wave and b-wave results in Cone-ERG. **(A)** Comparison of a-wave results in Cone-ERG among groups before modeling; **(B)** Comparison of b-wave results in Cone-ERG among groups before modeling; **(C)** Comparison of a-wave results in Cone-ERG among groups 4 weeks after modeling; **(D)** Comparison of b-wave results in Cone-ERG among groups 4 weeks after modeling; **(E)** Comparison of a-wave results in Cone-ERG among groups after 8 weeks of treatment; **(F)** Comparison of b-wave results in Cone-ERG among groups after 8 weeks of treatment. Cone-ERG: Cone cell response in light adaptation; NC: Normal control; LIM: Lens-induced myopia; DHA: Docosahexaenoic acid; BE: Bilberry extract. ns *p* > 0.05, *p < 0.05, **p < 0.01, ***p < 0.001.

The results of Cone-ERG indicated that under light-adapted conditions with LA 10 cd*s/m2 stimulation, there were no significant differences in a-waves and b-waves between the groups before modeling (Figures 7A,B). Four weeks after modeling, compared to the NC group (a-wave 8.55 ± 1.40 μV; b-wave 13.21 ± 4.22 μV), the Cone-ERG a-waves and b-waves of the right eye decreased in the LIM (a-wave 5.35 ± 0.94 μV; b-wave 9.80 ± 3.05 μV), LIM + DHA + BE (a-wave 5.39 ± 0.91 μV; b-wave 9.62 ± 2.94 μV), LIM + DHA (a-wave 5.40 ± 0.97 μV; b-wave 9.44 ± 3.00 μV), and LIM + BE (a-wave 5.44 ± 0.91 μV; b-wave 10.02 ± 2.98 μV) groups (Figures 7C,D, a-wave, *p* < 0.05; b-wave, *p* < 0.05). After 8 weeks of treatment, compared to the LIM group (a-wave 5.34 ± 0.94 μV; b-wave 9.84 ± 3.20 μV), the Cone-ERG a-waves and b-waves of the right eye increased in the LIM + DHA + BE (a-wave 8.59 ± 1.05 μV; b-wave 11.60 ± 3.92 μV), LIM + DHA (a-wave 7.04 ± 1.00 μV; b-wave 11.12 ± 3.80 μV), and LIM + BE (a-wave 7.03 ± 1.10 μV; b-wave 10.92 ± 3.09 μV) groups (Figures 7E,F, a-wave, *p* < 0.05; b-wave, *p* < 0.05). Additionally, OPS measurements showed no significant differences in light-adapted OPS waves before modeling, 4 weeks after modeling, and 8 weeks after treatment (Figures 6D–F). Max-ERG, OPS and Cone-ERG assays in the left eyes of guinea pigs in all groups were not significantly different at 4 weeks after modeling and 8 weeks after treatment (Supplementary Figures S3A–F, S4A–D).

## 4 Discussion

Myopia is the most common ocular disorder worldwide, particularly in Asia, where it represents a major cause of visual impairment. Consequently, recent studies have increasingly focused on decelerating the progression of myopia and mitigating its impact on children. Previous studies have highlighted the health benefits of DHA, such as its antioxidant and neuroprotective effects (30, 31). Recent human studies have found that DHA can delay the progression of myopia through its effects on blood vessels and blood flow (32, 33). In addition, decreased scleral DHA levels were observed in myopic guinea pigs (21). Additionally, it has been shown (34) that children must consume 20 mg/kg/day of DHA to maintain health; thus, the DHA intake of most children is insufficient, which can exacerbate the progression of myopia. Thus, DHA supplementation is a potential strategy to control the development of myopia among children. BE exhibited antioxidant and anti-inflammatory effects in several animal studies (21, 35). Moreover, recent studies have also discovered that BE can reduce blood pressure (36) and protect the eye by repairing the corneal epithelium and alleviating eye fatigue (26, 37, 38). Myopia reduces choroidal blood flow, but BE can improve both retinal and choroidal blood flow (39, 40), therefore, it can be an effective method for the prevention and control of myopia. DHA algal oil and BE are compatible and can be combined to improve their potency.

In this study, we administered 100 mg/kg/day of DHA and 16.5 mg/kg/day of BE to the LIM + DHA + BE group and found that the oral mixture of DHA and BE significantly inhibited the development of LIM in guinea pigs. After 8 weeks of treatment, compared to the LIM group, the LIM + DHA + BE group showed a greater reduction in myopic refractive error and shortening of the axial length. Recently, the clinical significance of ChT has gained increasing attention. The choroid, located between the retina and the sclera, is composed of blood vessels and stromal tissues. ChT can directly reflect the changes associated with myopia (41, 42). Additionally, the CVI is considered a more reliable index for assessing the vascular structure of the choroid (43). Recent studies have used CVI to evaluate the effect of myopia on choroidal blood flow in guinea pigs (44–48). This study employed ChT in conjunction with CVI to assess the effects of the DHA and BE combination on the choroid of myopic guinea pigs. After 8 weeks of treatment, compared to the LIM group, the ChT increased in the LIM + DHA + BE and LIM + DHA groups. The increase was particularly significant in the LIM + DHA + BE group. Similarly, there was a significant increase in the CVI of the LIM + DHA + BE group, while the CVI of the LIM + DHA and LIM + BE groups showed no significant changes.

Previous studies have indicated that scleral hypoxia of myopic eyes induces the differentiation of muscle fiber bundles, thereby reducing scleral collagen content and causing scleral thickening, which in turn leads to the development of myopia (49). In guinea pigs, increased choroidal blood flow and reduced scleral hypoxia effectively decelerated the progression of myopia (50). Our findings also demonstrated that the combination of DHA and BE can enhance ChT and ChBp. Compared to guinea pigs with thinner choroids, those with thicker choroids were at lower risk of myopia. A thicker choroid with greater blood flow provides stronger oxygenation to the adjacent sclera, reducing the likelihood of hypoxia and slowing the progression of myopia (51). Previous studies have indicated that patients with high myopia show decreased density of retinal cone cells, rod cells, and bipolar cells in the posterior pole in ERG, reflecting the reduced amplitudes of a-waves and b-waves. The a-wave represents the function of cone cells under light-adapted conditions and rod cells under dark-adapted conditions, while the b-wave represents the function of bipolar cells connected to cone cells. OPS waves represent the function of non-spiking cells. No significant changes were observed in the morphology, distribution, or number of other neural cells (51). Compared to human retinas, guinea pigs have a well-developed retina with a similar distribution of retinal structural cells. Cone-ERG and Max-ERG demonstrated that myopic eyes exhibit functional impairment in cone cells and rod cells, respectively. Due to the continuous stretching caused by the elongation of the myopic eyeball, retinal degenerative changes are at a greater risk of tensile forces, resulting in a significant decrease in the density of cone and rod cells. This effect is reflected in the results of Max-ERG and Cone-ERG. Consistently, 4 weeks after modeling, the a-waves and b-waves in the right eyes of guinea pigs were decreased in the LIM, LIM + DHA, LIM + BE, and LIM + DHA + BE groups compared to the NC group. After 8 weeks of treatment, ERG assessments, including Max-ERG and Cone-ERG tests, showed that the a-waves and b-waves improved in the right eyes of the LIM + DHA + BE, LIM + DHA, and LIM + BE groups compared to the LIM group. Notably, the recovery was greater in the LIM + DHA + BE group than in the LIM + DHA and LIM + BE groups. ERG demonstrated significant recovery in the function of cone cells, rod cells, and bipolar cells. DHA and BE restored the cellular morphology and density of cone and rod cells, thereby enhancing a-waves and b-waves. The lack of statistically significant changes in OPS waves before and after modeling indicates that myopia had a minimal impact on the function of non-spiking cells in guinea pigs; thus, no significant differences were observed before and after treatment.

## 5 Conclusion

This study demonstrated that the combination of DHA and BE can inhibit the progression of myopia and prevent the elongation of the ocular axis in a defocus-induced high myopia guinea pig model. The treatment effectively decelerated the decrease in ChT and ChBP, enhanced the CVI, and increased the activity of cone cells, rod cells, and bipolar cells, subsequently improving the a-waves and b-waves in Max-ERG and Cone-ERG. In summary, the combination of DHA and BE inhibited the progression of myopia in guinea pigs. DHA and BE also restored myopia-induced changes in choroidal structure and vascular system. The significant inhibition of myopia progression in the guinea pig model suggests that DHA and BE supplementation may protect against myopia in humans.

## Data Availability

The original contributions presented in the study are included in the article/Supplementary material, further inquiries can be directed to the corresponding author/s.

## References

[1] Zhu M-H Lin T-N Lin J-H Wen Q. Myopia among children and adolescents: an epidemiological study in Fuzhou City. Front Pediatr. (2023) 11:1161329. doi: 10.3389/fped.2023.1161329, PMID: 37384308 PMC10293673

[2] Zhong P Liu Y Ma N Dang J Shi D Cai S. Combined effect of outdoor time and other modifiable factors on myopia incidence among children and adolescents −9 PLADs, China, 2020. China CDC Wkly. (2024) 6:151–6. doi: 10.46234/ccdcw2024.031, PMID: 38495591 PMC10937186

[3] Eppenberger LS Grzybowski A Schmetterer L Ang M. Myopia control: are we ready for an evidence based approach? Ophthalmol Ther. (2024) 13:1453–77. doi: 10.1007/s40123-024-00951-w, PMID: 38710983 PMC11109072

[4] Ho C-L Wu W-F Liou YM. Dose-response relationship of outdoor exposure and myopia indicators: a systematic review and Meta-analysis of various research methods. Int J Environ Res Public Health. (2019) 16:2595. doi: 10.3390/ijerph16142595, PMID: 31330865 PMC6678505

[5] Simonaviciute D Gelzinis A Kapitanovaite L Grzybowski A Zemaitiene R. Myopia control in Caucasian children with 0.01% atropine eye drops: 1-year follow-up study. Medicina (Kaunas). (2024) 60:1022. doi: 10.3390/medicina60071022, PMID: 39064451 PMC11279162

[6] Gong Q Janowski M Luo M Wei H Chen B Yang G. Efficacy and adverse effects of atropine in childhood myopia: a Meta-analysis. JAMA Ophthalmol. (2017) 135:624–30. doi: 10.1001/jamaophthalmol.2017.1091, PMID: 28494063 PMC5710262

[7] Hui W Xiao-Feng H Song-Guo L Jing-Jing W Xuan H Yong T. Application of orthokeratology on myopia control and its effect on ocular surface and meibomian gland function in Chinese myopic adolescents. Front Med (Lausanne). (2022) 9:979334. doi: 10.3389/fmed.2022.979334, PMID: 36569150 PMC9772008

[8] Kam KW Yung W Li GKH Chen LJ Young AL. Infectious keratitis and orthokeratology lens use: a systematic review. Infection. (2017) 45:727–35. doi: 10.1007/s15010-017-1023-2, PMID: 28534320

[9] Lin T Su L Lin J Qiu H. Study on the optic nerve Fiber layer thickness and changes in blood flow in myopic children. Int J Gen Med. (2021) 14:3287–93. doi: 10.2147/IJGM.S317476, PMID: 34267547 PMC8276819

[10] Zhou X Zhang S Yang F Yang Y Huang Q Huang C. Decreased choroidal blood perfusion induces myopia in Guinea pigs. Invest Ophthalmol Vis Sci. (2021) 62:30. doi: 10.1167/iovs.62.15.30, PMID: 34967855 PMC8740532

[11] Liu Y Wang L Xu Y Pang Z Mu G. The influence of the choroid on the onset and development of myopia: from perspectives of choroidal thickness and blood flow. Acta Ophthalmol. (2021) 99:730–8. doi: 10.1111/aos.14773, PMID: 33550704

[12] Yang J Ouyang X Fu H Hou X Liu Y Xie Y. Advances in biomedical study of the myopia-related signaling pathways and mechanisms. Biomed Pharmacother. (2022) 145:112472. doi: 10.1016/j.biopha.2021.112472, PMID: 34861634

[13] Yu Z Zhong A Zhao X Li D Duan J. Efficacy and safety of different add power soft contact lenses on myopia progression in children: a systematic review and Meta-analysis. Ophthalmic Res. (2022) 65:398–416. doi: 10.1159/000523675, PMID: 35226916

[14] Xu X Liu N Yu W. No evidence of an association between genetic factors affecting response to vitamin a supplementation and myopia: a Mendelian randomization study and Meta-analysis. Nutrients. (2024) 16:1933. doi: 10.3390/nu16121933, PMID: 38931287 PMC11206965

[15] Yoshida T Takagi Y Igarashi-Yokoi T Ohno-Matsui K. Efficacy of lutein supplements on macular pigment optical density in highly myopic individuals: a randomized controlled trial. Medicine (Baltimore). (2023) 102:e33280. doi: 10.1097/MD.0000000000033280, PMID: 36961139 PMC10036027

[16] Li M Tan C-S Foo L-L Sugianto R Toh JY Sun C-H. Dietary intake and associations with myopia in Singapore children. Ophthalmic Physiol Opt. (2022) 42:319–26. doi: 10.1111/opo.12929, PMID: 34862645

[17] Chamarty S Gupta SK Dhakal R Verkicharla PK. Is there any association between nutrition and myopia? A systematic review. Optom Vis Sci. (2023) 100:475–85. doi: 10.1097/OPX.0000000000002035 37399226

[18] Dicks LMT. How important are fatty acids in human health and can they be used in treating diseases? Gut Microbes. (2024) 16:2420765. doi: 10.1080/19490976.2024.2420765, PMID: 39462280 PMC11520540

[19] Troesch B Eggersdorfer M Laviano A Rolland Y Smith AD Warnke I. Expert opinion on benefits of long-chain Omega-3 fatty acids (DHA and EPA) in aging and clinical nutrition. Nutrients. (2020) 12:2555. doi: 10.3390/nu12092555, PMID: 32846900 PMC7551800

[20] Xue CC Li H Dong XX Yu M Soh ZD Chong CCY. Omega-3 polyunsaturated fatty acids as a protective factor for myopia. Am J Ophthalmol. (2024) 268:368–4377. doi: 10.1016/j.ajo.2024.08.041, PMID: 39244001 PMC11606739

[21] Pan M Zhao F Xie B Wu H Zhang S Ye C. Dietary ω-3 polyunsaturated fatty acids are protective for myopia. Proc Natl Acad Sci USA. (2021) 118:e2104689118. doi: 10.1073/pnas.2104689118, PMID: 34675076 PMC8639353

[22] Mori K Kuroha S Hou J Jeong H Ogawa M Ikeda S-I. Lipidomic analysis revealed n-3 polyunsaturated fatty acids suppressed choroidal thinning and myopia progression in mice. FASEB J. (2022) 36:e22312. doi: 10.1096/fj.202101947R, PMID: 35532744

[23] Sinclair AJ Wang Y Li D. What is the evidence for dietary-induced DHA deficiency in human brains? Nutrients. (2022) 15:161. doi: 10.3390/nu15010161, PMID: 36615819 PMC9824463

[24] Utami AT Muzaahim A. Honey and bilberry fruit extract to reduce myopia: A case study in Indonesia. J Clin Ophthalmol Eye Disord. (2024) 8:1.

[25] Vaneková Z Rollinger JM. Bilberries: curative and miraculous - a review on bioactive constituents and clinical research. Front Pharmacol. (2022) 13:909914. doi: 10.3389/fphar.2022.909914, PMID: 35847049 PMC9277355

[26] Kosehira M Machida N Kitaichi N. A 12-week-long intake of bilberry extract (*Vaccinium myrtillus* L.) improved objective findings of ciliary muscle contraction of the eye: a randomized, double-blind, placebo-controlled, parallel-group comparison trial. Nutrients. (2020) 12:600. doi: 10.3390/nu12030600, PMID: 32106548 PMC7146147

[27] Yu WY Chan LYL Chung A Lee PH Woo GC. Bilberry-containing supplements on severe dry eye disease in young and middle-aged adults: a 3-month pilot analysis. Front Nutr. (2023) 10:1061818. doi: 10.3389/fnut.2023.1061818, PMID: 36742436 PMC9892183

[28] Prieto Martínez A Coutiño Diaz M Anaya Romero L Ali Redha A Zare R Ventura Hernandez S. Effects of Vaccinium berries (blueberries, cranberries and bilberries) on oxidative stress, inflammation, exercise performance, and recovery - a systematic review. Food Funct. (2024) 15:444–59. doi: 10.1039/d3fo04435a, PMID: 38165220

[29] Li B Wang L Bai W Chen W Chen F Shu C. Anthocyanins: chemistry, processing and bioactivity. Singapore: Springer Nature (2021).

[30] Díaz M Mesa-Herrera F Marín R. DHA and its elaborated modulation of antioxidant defenses of the brain: implications in aging and AD neurodegeneration. Antioxidants (Basel). (2021) 10:907. doi: 10.3390/antiox10060907, PMID: 34205196 PMC8228037

[31] Lafuente M Rodríguez González-Herrero ME Romeo Villadóniga S Domingo JC. Antioxidant activity and neuroprotective role of docosahexaenoic acid (DHA) supplementation in eye diseases that can Lead to blindness: a narrative review. Antioxidants (Basel). (2021) 10:386. doi: 10.3390/antiox10030386, PMID: 33807538 PMC8000043

[32] Zhou Z Li S Yang Q Yang X Liu Y Hao K. Association of n-3 polyunsaturated fatty acid intakes with juvenile myopia: a cross-sectional study based on the NHANES database. Front Pediatr. (2023) 11:1122773. doi: 10.3389/fped.2023.1122773, PMID: 37138572 PMC10150007

[33] Zhang AC Singh S Craig JP Downie LE. Omega-3 fatty acids and eye health: opinions and self-reported practice behaviors of optometrists in Australia and New Zealand. Nutrients. (2020) 12:1179. doi: 10.3390/nu12041179, PMID: 32331489 PMC7230711

[34] Bailey ADL Fulgoni Iii VL Shah N Patterson AC Gutierrez-Orozco F Mathews RS. Nutrient intake adequacy from food and beverage intake of US children aged 1-6 years from NHANES 2001-2016. Nutrients. (2021) 13:827. doi: 10.3390/nu13030827, PMID: 33802295 PMC8002201

[35] Bayazid AB Chun EM Al Mijan M Park SH Moon S-K Lim BO. Anthocyanins profiling of bilberry (*Vaccinium myrtillus* L.) extract that elucidates antioxidant and anti-inflammatory effects. Food Agric Immunol. (2021) 32:713–26. doi: 10.1080/09540105.2021.1986471

[36] Grohmann T Litts C Horgan G Zhang X Hoggard N Russell W. Efficacy of bilberry and grape seed extract supplement interventions to improve glucose and cholesterol metabolism and blood pressure in different populations-a systematic review of the literature. Nutrients. (2021) 13:1692. doi: 10.3390/nu13051692, PMID: 34067538 PMC8156535

[37] Ooe E Yako T Kuse Y Sogon T Nakamura S Shimazawa M. The protective effects of bilberry extract and its main constituents against blue light-emitting diode (LED) light-induced damage in human corneal epithelial cells. Jpn Health Nutr Assoc. (2020) 14:120. doi: 10.20618/jhnfa.17.1_18

[38] Szumny D Kucharska AZ Czajor K Bernacka K Ziółkowska S Krzyżanowska-Berkowska P. Extract from *Aronia melanocarpa*, *Lonicera caerulea*, and *Vaccinium myrtillus* improves near visual acuity in people with presbyopia. Nutrients. (2024) 16:926. doi: 10.3390/nu16070926, PMID: 38612968 PMC11013737

[39] Xing Y Liang S Zhao Y Yang S Ni H Li H. Protection of *Aronia melanocarpa* fruit extract from sodium-iodate-induced damages in rat retina. Nutrients. (2021) 13:4411. doi: 10.3390/nu13124411, PMID: 34959962 PMC8703977

[40] Wang Y Zhao L Lu F Yang X Deng Q Ji B. Retinoprotective effects of bilberry anthocyanins via antioxidant, anti-inflammatory, and anti-apoptotic mechanisms in a visible light-induced retinal degeneration model in pigmented rabbits. Molecules. (2015) 20:22395–410. doi: 10.3390/molecules201219785, PMID: 26694327 PMC6332335

[41] Prousali E Dastiridou A Ziakas N Androudi S Mataftsi A. Choroidal thickness and ocular growth in childhood. Surv Ophthalmol. (2021) 66:261–75. doi: 10.1016/j.survophthal.2020.06.008, PMID: 32634443

[42] Spaide RF. The choroid In: Spaide RF Ohno-Matsui K Yannuzzi LA, editors. Pathologic myopia. Cham: Springer International Publishing (2021). 139–59.

[43] Agrawal R Ding J Sen P Rousselot A Chan A Nivison-Smith L. Exploring choroidal angioarchitecture in health and disease using choroidal vascularity index. Prog Retin Eye Res. (2020) 77:100829. doi: 10.1016/j.preteyeres.2020.100829, PMID: 31927136

[44] Wan T Shi W Liang R Li T Li B Zhou X. VEGFA may be a potential marker of myopic choroidal thickness and vascular density changes. Sci Rep. (2024) 14:20514. doi: 10.1038/s41598-024-70616-y, PMID: 39227639 PMC11372119

[45] Xiang A He H Li A Meng X Luo Y Luo Y. Changes in choroidal thickness and blood flow in response to form deprivation-induced myopia and repeated low-level red-light therapy in Guinea pigs. Ophthalmic Physiol Opt. (2024) 1:404. doi: 10.1111/opo.13404, PMID: 39367704

[46] Liu Y Zhu M Yan X Li M Xiang Y. The effect of repeated low-level red-light therapy on myopia control and choroid. Transl Vis Sci Technol. (2024) 13:29. doi: 10.1167/tvst.13.10.29, PMID: 39432402 PMC11498649

[47] Han R Chang W Ding X Jiang R Chang Q Xu G. The choroid vascular index and its association with visual acuity in children and young adults with high myopia. Eye (Lond). (2023) 37:2542–7. doi: 10.1038/s41433-022-02369-9, PMID: 36539600 PMC10397268

[48] Yang Y Chen M Yao X Wang J Shi J Wang Y. Choroidal blood perfusion could predict the sensitivity of myopia formation in Guinea pigs. Exp Eye Res. (2023) 232:109509. doi: 10.1016/j.exer.2023.109509, PMID: 37247833

[49] Wu H Chen W Zhao F Zhou Q Reinach PS Deng L. Scleral hypoxia is a target for myopia control. Proc Natl Acad Sci U S A. (2018) 115:E7091–E 7100. doi: 10.1073/pnas.1721443115 29987045 PMC6064999

[50] Che D Qiao D Cao Y Zhang Y Zhou Q Tong S. Changes in choroidal hemodynamics of form-deprivation myopia in Guinea pigs. Biochem Biophys Res Commun. (2024) 692:149348. doi: 10.1016/j.bbrc.2023.149348, PMID: 38064999

[51] Kader MA. Electrophysiological study of myopia. Saudi J Ophthalmol. (2012) 26:91–9. doi: 10.1016/j.sjopt.2011.08.002, PMID: 23960975 PMC3729644

