# Supplementary material for: Protective effects of docosahexaenoic acid combined with bilberry extract on myopic Guinea pigs
Source: Front Med (Lausanne). 2024 Dec 17;11:1502612. doi: 10.3389/fmed.2024.1502612 (PMC11685150; doi:10.3389/fmed.2024.1502612)
Supplement: Supplementary file 1 [file Data_Sheet_1.docx]

Supplementary Material

# Supplementary Figures and Tables


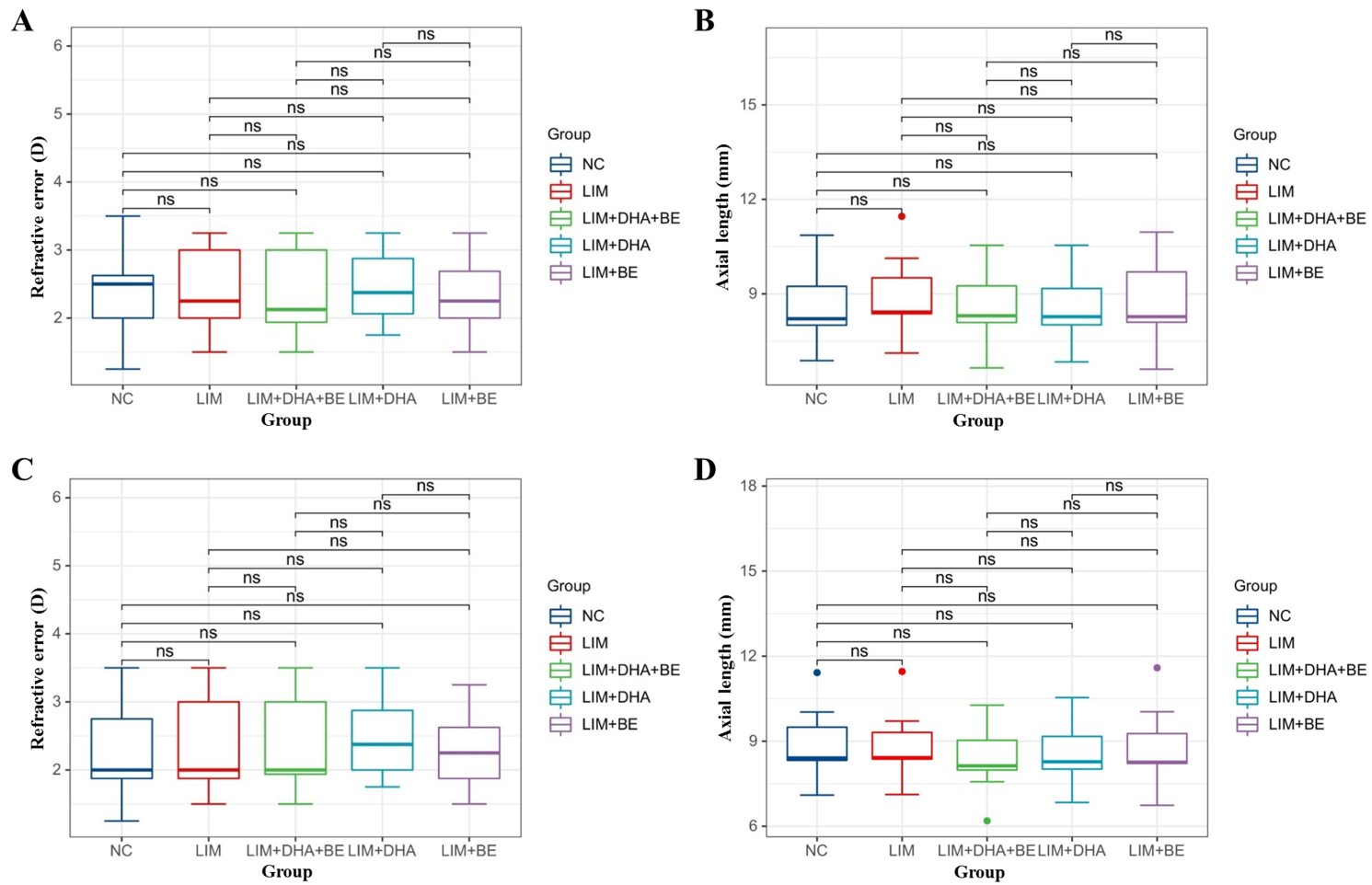


**Figure S1.** Comparison of refractive error and eye axis test results in the left eye 4 weeks after modeling and 8 weeks after treatment. A. Comparison of left eye refraction 4 weeks after modeling; B. Comparison of the ocular axis of the left eye 4 weeks after modeling; C. Comparison of refraction in the left eye 8 weeks after treatment; D. Comparison of left eye axes 8 weeks after treatment. NC: Normal control; LIM: Lens-induced myopia; DHA: Docosahexaenoic acid; BE: Bilberry extract. ns P > 0.05.


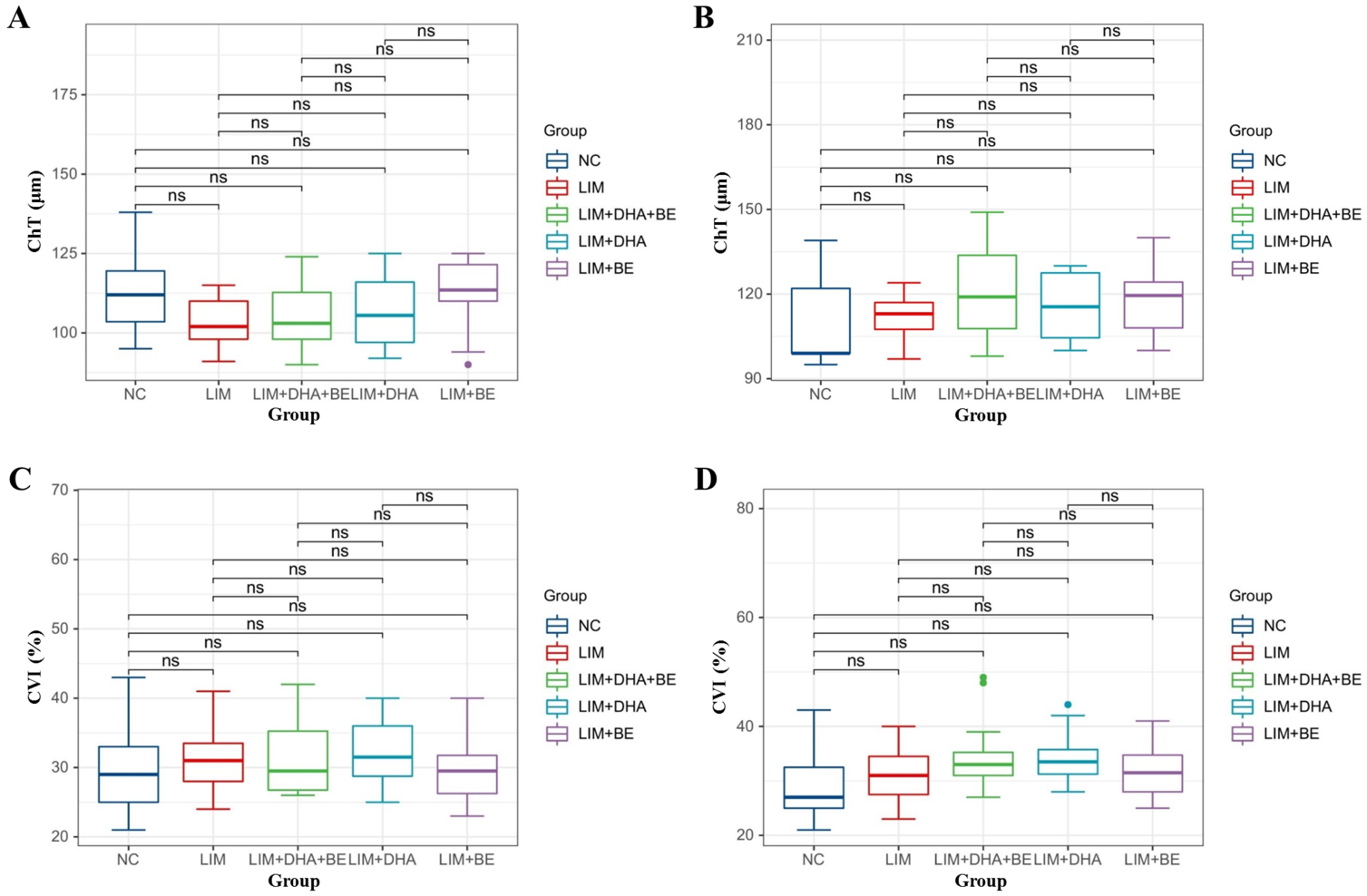


**Figure S2.** Comparison of ChT and CVI test results in the left eye 4 weeks after modeling and 8 weeks after treatment. A. Comparison of ChT in the left eye 4 weeks after modeling; B. Comparison of ChT in the left eye 8 weeks after treatment; C. Comparison of CVI in the left eye 4 weeks after modeling; D. Comparison of CVI in the left eye 8 weeks after treatment. ChT: Choroidal thickness; CVI: Choroidal vascularity index; NC: Normal control; LIM: Lens-induced myopia; DHA: Docosahexaenoic acid; BE: Bilberry extract. ns P > 0.05.


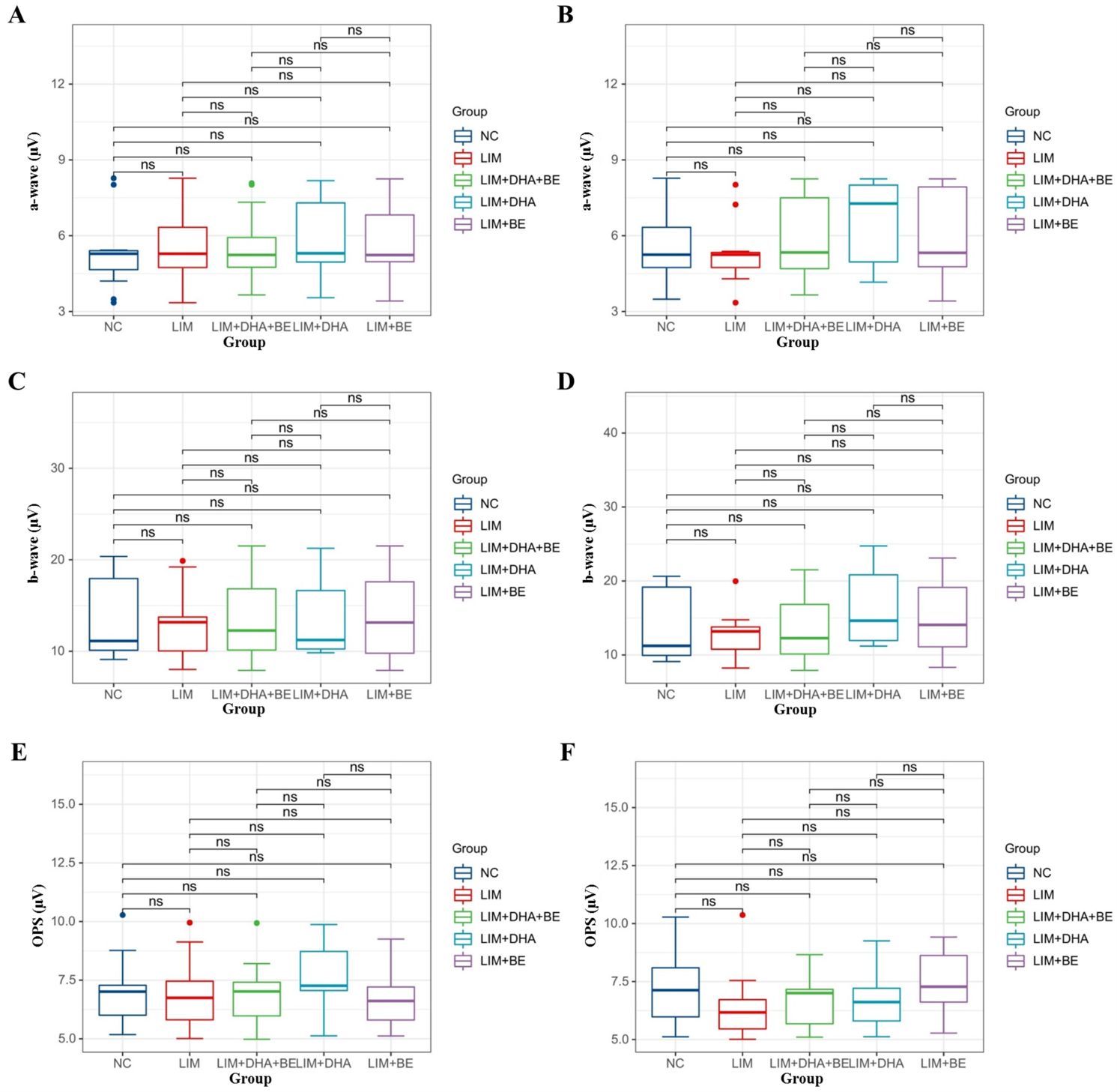


**Figure S3.** Comparison of Max-ERG ab and OPS waves in the left eye 4 weeks after modeling and 8 weeks after treatment. A. Comparison of Max-ERG a wave in the left eye 4 weeks after modeling; B. Comparison of Max-ERG a wave in the left eye 8 weeks after treatment; C. Comparison of Max-ERG b-wave in the left eye 4 weeks after modeling; D. Max-ERG b-wave comparison of the left eye 8 weeks after treatment; E. Comparison of OPS wave in the left eye 4 weeks after modeling; F. Comparison of OPS waves in the left eye 8 weeks after treatment. Max-ERG: Maximal mixed response in dark adaptation; OPS: oscillatory potentials in both dark and light adaptation; NC: Normal control; LIM: Lens-induced myopia; DHA: Docosahexaenoic acid; BE: Bilberry extract. ns P > 0.05.


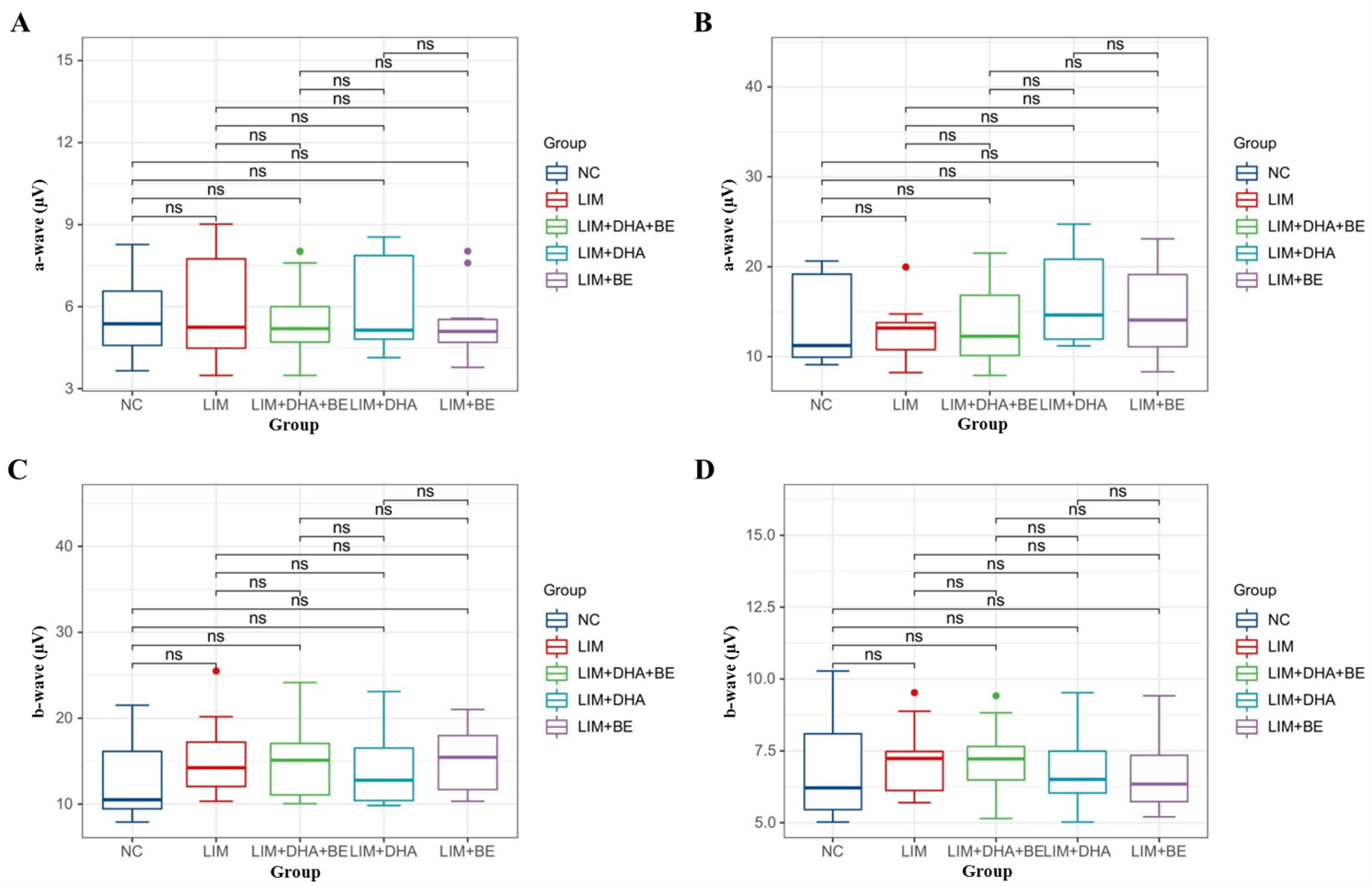


**Figure S4.** Comparison of Cone-ERG a-wave and Cone-ERG b-wave in the left eye 4 weeks after modeling and 8 weeks after treatment. A. Comparison of Cone-ERG a wave in the left eye 4 weeks after modeling; B. Comparison of Cone-ERG a wave in the left eye 8 weeks after treatment; C. Cone-ERG b-wave comparison of the left eye 4 weeks after modeling; D. Cone-ERG b-wave comparison of the left eye 8 weeks after modeling. Cone-ERG: Cone cell response in light adaptation; OPS: oscillatory potentials in both dark and light adaptation; NC: Normal control; LIM: Lens-induced myopia; DHA: Docosahexaenoic acid; BE: Bilberry extract. ns P > 0.05.

Table S1. Comparison of ChT and CVI at 8 weeks of treatment.

| Project | 8 weeks of treatment ChT (μm) | 8 weeks of treatment CVI (%) |
| --- | --- | --- |
| NC | 113.82 ± 13.6 | 30.18 ± 6.63 |
| LIM | 89.00 ± 10.37 | 22.73 ± 4.43 |
| LIM+BE | 97.30 ± 9.52 *** | 24.07 ± 3.62 |
| LIM+DHA | 94.07 ± 10.25 *** | 23.70 ± 3.89 |
| LIM+DHA+BE | 98.08 ± 9.62 *** | 27.17 ± 5.46 * |
| F-value | 56.747 | 64.61 |
| P-value | < 0.001 | < 0.001 |

*Indicates statistically significant difference compared with LIM group; ChT: Choroidal thickness; CVI: Choroidal vascularity index; NC: Normal control; LIM: Lens-induced myopia; DHA: Docosahexaenoic acid; BE: Bilberry extract. *P < 0.05, ***P < 0.001. Mean ± SD.

**Preparation of DHA + Bilberry Extract:**

Composition of the mixed sample (soft capsule contents): primarily bilberry extract and DHA algal oil. Recommended dosage is 1 g/d based on an adult weight of 60 kg. The bilberry extract (70% ethanol extraction) has an anthocyanin content of 10%. The effective dosage of anthocyanins is 200 mg/d, equivalent to 2 g/d of bilberry extract. Based on an adult weight of 60 kg, this corresponds to 3.3 mg/kg. DHA algal oil (containing 35% DHA) has an effective dosage of 300 mg/d, equivalent to 0.86 g/d of DHA algal oil. Based on an adult weight of 60 kg, this corresponds to 14.3 mg/kg. The equivalent dosage for guinea pigs compared to humans is 5 times.

The preparation process is as follows:

1. Raw and auxiliary materials: Collect all raw and auxiliary materials according to the production plan, and they must be inspected and approved by the quality control department before use.
2. Weighing: Weigh the required amounts of bilberry extract, DHA algal oil, beeswax, gelatin, glycerin, and purified water according to the formula.
3. Gelatinization
   **3.1** Add the specified amount of purified water and glycerin into the gelatinization tank while starting the stirring paddle. When the temperature in the gelatinization tank reaches 70°C, add the specified amount of gelatin, continue heating to 75°C, and maintain stirring for about 1 hour until the gelatin is completely dissolved, resulting in a gel liquid mixture.
   **3.2** Vacuum extraction: Apply vacuum to the gel liquid mixture (vacuum degree -0.06 Mpa) until there are no air bubbles, approximately 30 minutes, then release the vacuum.
   **3.3** Gel liquid filtration: Filter the degassed gel liquid using a 100-mesh stainless steel sieve.
   **3.4** Standing: After filtration, place the gel liquid in a warm storage tank and let it stand for 4-5 hours while maintaining a temperature of 65°C, ready for use.
4. Ingredient mixing
   **4.1** Wax melting: Heat the specified amount of beeswax to 70°C until fully melted, then slowly add it to the DHA algal oil while stirring. Continue stirring until uniform, resulting in an oil-wax liquid.
   **4.2** Mixing: Mix the specified amounts of bilberry extract, lutein, and zeaxanthin in a three-dimensional mixer for 20 minutes until uniform; add the mixed powder to the oil-wax liquid while stirring, and continue stirring for 30 minutes after all is added; pass through a colloid mill 2-3 times for uniformity, resulting in a mixed liquid.
   **4.3** Filtration and vacuum extraction: Filter the mixed liquid using a 100-mesh sieve and apply vacuum at -0.06 MPa to remove air bubbles, resulting in the capsule contents.
5. Tablet pressing: Before tablet pressing, check the equipment to ensure it is functioning properly. First, use paraffin oil to adjust the tablet press, measure the tablet weight to meet quality requirements, and check the appearance (consistency of thickness on both sides of the capsule, firm joints, etc.). Once all checks are qualified, normal tablet pressing can commence. The capsule contents and gel liquid are passed through the soft capsule machine to form soft capsules, with a filling amount of 0.5 g/capsule. During the pressing process, check the tablet weight approximately every 10 minutes to ensure the weight variation is within acceptable limits, and continuously check the capsule appearance and shape for any leaks. Pay close attention to the operation of the equipment during production.
   Tablet pressing workshop: Indoor temperature 18-26°C, relative humidity 45-55%.
6. Shaping: The pressed capsules enter the shaping drum, where they are shaped with cold air (18-26°C) for 2-3 hours.
   Shaping workshop: Indoor temperature 18-26°C, relative humidity 45-55%.
7. Drying: Transfer the appropriately hardened soft capsules to a drying room for ventilation and drying, flipping the capsules every 2 hours. The drying time is approximately 20-24 hours until the capsule moisture content is 10-12%.
   Drying room: Indoor temperature 21-26°C, relative humidity 20-40%.
8. Light inspection: Place the dried soft capsules on a light inspection machine, manually selecting out any malformed capsules, flat capsules, thin-walled capsules, and those with inconsistent sizes. Qualified soft capsules are set aside for packaging.
9. Packaging
   **9.1** Inner packaging: Place qualified soft capsules into bottles. The specification is 30 capsules/bottle. The inner packaging uses plastic bottles, and the quality standards for plastic bottles must comply with the "National Food Safety Standard for Plastic Materials and Products for Food Contact" (GB 4806.7-2016).
   **9.2** Outer packaging: Carton packaging. The outer packaging uses corrugated cartons, which must comply with GB/T 6543.
10. Inspection and warehousing: Conduct inspections according to product technical requirements, and qualified products are stored in the warehouse.
